# Comparative Omics and Trait Analyses of Marine *Pseudoalteromonas* Phages Advance the Phage OTU Concept

**DOI:** 10.3389/fmicb.2017.01241

**Published:** 2017-07-06

**Authors:** Melissa B. Duhaime, Natalie Solonenko, Simon Roux, Nathan C. Verberkmoes, Antje Wichels, Matthew B. Sullivan

**Affiliations:** ^1^Department of Ecology and Evolutionary Biology, University of Michigan, Ann ArborMI, United States; ^2^Department of Microbiology, The Ohio State University, ColumbusOH, United States; ^3^Department of Biological Sciences, Border Biomedical Research Center, University of Texas at El Paso, El PasoTX, United States; ^4^Biologische Anstalt Helgoland, Alfred Wegener Institute, Helmholtz Centre for Polar and Marine ResearchHelgoland, Germany; ^5^Department of Civil, Environmental, and Geodetic Engineering, The Ohio State University, ColumbusOH, United States

**Keywords:** phage, marine microbiology, particle-associated, comparative genomics, *Pseudoalteromonas*

## Abstract

Viruses influence the ecology and evolutionary trajectory of microbial communities. Yet our understanding of their roles in ecosystems is limited by the paucity of model systems available for hypothesis generation and testing. Further, virology is limited by the lack of a broadly accepted conceptual framework to classify viral diversity into evolutionary and ecologically cohesive units. Here, we introduce genomes, structural proteomes, and quantitative host range data for eight *Pseudoalteromonas* phages isolated from Helgoland (North Sea, Germany) and use these data to advance a genome-based viral operational taxonomic unit (OTU) definition. These viruses represent five new genera and inform 498 unaffiliated or unannotated protein clusters (PCs) from global virus metagenomes. In a comparison of previously sequenced *Pseudoalteromonas* phage isolates (*n* = 7) and predicted prophages (*n* = 31), the eight phages are unique. They share a genus with only one other isolate, *Pseudoalteromonas* podophage RIO-1 (East Sea, South Korea) and two *Pseudoalteromonas* prophages. Mass-spectrometry of purified viral particles identified 12–20 structural proteins per phage. When combined with 3-D structural predictions, these data led to the functional characterization of five previously unidentified major capsid proteins. Protein functional predictions revealed mechanisms for hijacking host metabolism and resources. Further, they uncovered a hybrid sipho-myovirus that encodes genes for Mu-like infection rarely described in ocean systems. Finally, we used these data to evaluate a recently introduced definition for virus populations that requires members of the same population to have >95% average nucleotide identity across at least 80% of their genes. Using physiological traits and genomics, we proposed a conceptual model for a viral OTU definition that captures evolutionarily cohesive and ecologically distinct units. In this trait-based framework, sensitive hosts are considered viral niches, while host ranges and infection efficiencies are tracked as viral traits. Quantitative host range assays revealed conserved traits within virus OTUs that break down between OTUs, suggesting the defined units capture niche and fitness differentiation. Together these analyses provide a foundation for model system-based hypothesis testing that will improve our understanding of marine copiotrophs, as well as phage–host interactions on the ocean particles and aggregates where *Pseudoalteromonas* thrive.

## Introduction

Microbes and their metabolic outputs impact diverse ecosystem functions (Falkowski et al., 2008) and viruses tune these microbial metabolisms through mortality, horizontal gene transfer, and host metabolic reprogramming (Fuhrman, 1999; Wommack and Colwell, 2000; Suttle, 2007; Breitbart, 2012; Brum and Sullivan, 2015). In the oceans, micron- to millimeter-sized particles (e.g., marine snow, fecal pellets, gelatinous exudates, and carcasses of zooplankton) are hotspots of this microbial metabolic activity (Azam, 1998), in particular heterotrophic activity (Ploug et al., 1999). The microbial processes that take place on and in ocean particles modulate the flux of organic matter, thereby impacting the efficiency of the biological carbon pump and ultimately the planetary climate system (Shanks and Trent, 1980; Ducklow et al., 2001). Viruses are present and active on these sinking particles (Bratbak et al., 1990; Proctor and Fuhrman, 1991; Weinbauer et al., 2009). Recently, depth-stratified viral community sequencing studies (viromes) have provided “genetic tracer” evidence that surface water viruses sink to the deep sea, which has been hypothesized to occur in association with particles (Brum and Sullivan, 2015; Brum et al., 2015; Hurwitz et al., 2015). Following on this, a recent study that examined the coupling between omics-based plankton community structure and surface ocean carbon export found that viruses are the best predictors of ocean carbon flux (Guidi et al., 2016). These recent reports highlight the need to revisit decades-old hypotheses about particle-adsorbed viruses (Proctor and Fuhrman, 1991). Further, they invite questions, such as what is the impact of sinking particle microcosms on virus and host biogeography, phage–host co-evolution, and predator–prey infection dynamics? And in turn, what are the impacts of these viral processes on the sinking rates of ocean particles and thereby the efficiency of the global biological carbon pump?

*Pseudoalteromonas* spp. (Gammaproteobacteria; Alteromonadales) are ideal hosts for developing a model particle-associated phage and host system. On the global scale, *Pseudoalteromonas* spp. are ubiquitous marine heterotrophs adapted to life on particles (Thomas et al., 2008), where they are highly represented and commonly constitute up to 20% of particle-associated (Fontanez et al., 2015) and particle-enriched (Smriga et al., 2016) microbial communities. Further, *Pseudoalteromonas* is the heterotrophic genus most strongly correlated with carbon export in the world’s oceans (Guidi et al., 2016). On the microscale, members of the genus have shown a strong and rapid chemotactic response toward dissolved organic matter plumes (Stocker et al., 2008), implicating them as model gradient-responding copiotrophs for the study of ocean particle ecology. Relationships with viruses are likely to impact the role of *Pseudoalteromonas* in the ocean particle habitat. In addition to the direct impact of host cell lysis on carbon flux, *Pseudoalteromonas* phages may impact the behavioral ecology of their microbial hosts in a manner that could modulate the magnitude of this effect: a filamentous *Pseudoalteromonas* phage has been shown to enhance the motility and chemotaxis of its infected host in culture (Yu et al., 2015).

Currently, phages with >95% average nucleotide identity (ANI) across at least 80% of their genes are assigned to a single phage population (Brum et al., 2015; Gregory et al., 2016), or as termed here, “phage OTU”—consistent with the 95% ANI cut-off proposed for microbial species (Konstantinidis and Tiedje, 2005). For phages, this concept has been in development for some time and has been supported by the stable spatial and temporal distribution of T4-like cyanomyophage isolates based on gene marker (Marston and Amrich, 2009) and full-genome analyses (Marston and Martiny, 2016), as well as genome-wide analyses of viral-tagged metagenomic contigs from wild virus populations (Deng et al., 2014). Most recently, a phylogenomic analysis of 142 marine T4-like cyanophages isolated on a single host (*Synechococcus* WH7803) observed that when this >95% threshold was applied, (i) recombination rates were greater within genotypic phage populations than between them, indicative of intra-population barriers to gene flow and (ii) different genes were under selection in the different populations, while the selection profile was conserved within populations (Gregory et al., 2016). While trends have emerged from genomic data to support a phage operational taxonomic unit (OTU) definition, these studies have not yet included physiological tests that would enable evaluation of fitness differences between genotypic populations.

To inform our understanding of the role of *Pseudoalteromonas* (PSA) phages in particle and ocean ecology, we sought to characterize the life history traits (e.g., host range, burst size, latent period), structural proteomes, and genomes of eight newly introduced PSA phages isolated offshore of the island Helgoland in the North Sea. These combined trait-based and comparative genomics analyses allowed us to test the validity of proposed sequence-based virus population delineations described above (Brum et al., 2015; Gregory et al., 2016). To do so, we considered *Pseudoalteromonas* hosts as viral niches and measured infection traits indicative of viral fitness in each niche. We hypothesized that if the currently proposed population (or phage OTU) definition were meaningful (i.e., capture evolutionarily and ecologically cohesive populations; Polz et al., 2006), trait-based differentiation would arise consistent with these genome-based boundaries. These analyses provide a baseline for understanding the ecological and evolutionary impact of viruses infecting *Pseudoalteromonas*, a model ocean particle-associated copiotrophic microbe.

## Materials and Methods

### Phage Harvesting and DNA Extraction

Phage and hosts were isolated from unfiltered, whole seawater in 1990 from the ‘Kabeltonne’ station near Helgoland, Germany (54.18 N 7.9 E), 55 km north of the German coast in the North Sea (Moebus, 1992; Wichels et al., 1998). In 2010, phages were recovered from liquid lysates stored at room temperature and hosts from glycerol stocks stored in liquid nitrogen. Host growth and infections were performed as described (Duhaime et al., 2011), with the exception of host growth media, which was altered to a “*Pseudoalteromonas* Zobell Media (PZM).” PZM was comprised of 50% of all components of the previously described Zobell Media, except sea salts remain at 25 g L^-1^. Host cultures were grown at 21°C and shaken at 150 rpm. Phage DNA was extracted using the Wizard PCR Prep DNA Purification Resin and Mini-columns (Promega, San Luis Obispo, CA, United States) per manufacturer provided protocol. Phage genomes, annotations, and associated metadata are publically available in the Joint Genome Institute’s Integrated Microbial Genomes (JGI-IMG) database with the following IMG Taxon IDs: 2582581227, 2582581228, 2582581229, 2582581230, 2582581231, 2582581232, 2582581235, 2622736497.

### Transmission Electron Microscopy

Concentrated viral lysates (>10^8^ viruses ml^-1^) were CsCl-purified (Duhaime et al., 2011) and 5 μl deposited on formvar coated 200 mesh copper grids (Electron Microscopy Sciences, Hatfield, PA, United States) that had been glow discharged for 3 min with a sputter coater (Hummer 6.2, Anatech, Union City, CA, United States). Grids were then stained with three drops of 0.02 μm-filtered 2% (w/v) uranyl acetate and for 30 s followed by three 10-s washes in ultra-pure water. All liquid was wicked away with filter paper to achieve negatively stained viral specimen. Grids were left to dry overnight in a desiccator at ambient temperature. Dry grids were visualized with a transmission electron microscope (Philips CM12, FEI, Hillsboro, OR, United States) at 80 kV accelerating voltage and 65,000–100,000 magnification. Micrographic images were collected using a Macrofire Monochrome CCD camera (Optronics, Goleta, CA, United States).

### Phage Infection Properties: One-Steps, Burst Sizes, Latent Periods

Before the one-step experiment was performed, the relationship between culture optical density (OD) and colony-forming units was established through a host growth curve. One-step experiments were performed in triplicate at targeted initial multiplicities of infection (MOI) of 0.1. Viruses were added to host cultures in mid-exponential (log linear) growth phase, whereby the host cell concentration was determined according to the OD-CFU correlation and the viruses were added accordingly to achieve the desired MOI (typically 10^7^ viruses added to 10^8^ host cells). No-phage controls were monitored in parallel. At *t* = 0, 15 min post-inoculation with viruses, cultures were diluted 1:100 in 50 ml PZM in a 250 ml glass Erlenmeyer flask and returned to the shaking incubator for the remainder of the experiment. At *t* = 0, the number of total viruses was quantified via plaque-forming units (PFUs) to determine the number of phages that contributed to subsequent infections. Sub-samples were taken every 20 min for 3 h and PFUs from free viruses (<0.2 μm filtrate) were quantified at each time point in duplicate using the agar-overlay method (Duhaime et al., 2011). The ends of the burst periods were determined where there was no significant change in the number of PFUs/ml from one time point to the next (Student’s *t*-test). The burst size was calculated as the difference in PFUs/ml before the initial rise and after the first burst divided by the number of initially infecting phage.

### Mass Spectroscopy-Based Structural Proteomics

Cesium chloride-purified phages were tryptically digested for 2D nano-LC-MS/MS analyses with an optimized Filter-Aided Sample Preparation kit (Expedeon, Inc., San Diego, CA, United States) (VerBerkmoes et al., 2009). MS/MS spectra were generated on a Velos OrbiTrap mass spectrometer (Thermo Scientific, Waltham, MA, United States), as described (Holmfeldt et al., 2013). To recruit peptides to the phage genomes, spectra were searched using SEQUEST against a database consisting of the annotated phage proteins, all possible phage ORFs > 30 aa in all six reading frames, and eukaryotic organisms (human and mouse) to use as indicators for false positives. Data analyses were performed as described (Holmfeldt et al., 2013). A normalized spectral abundance factor (NSAF; Paoletti et al., 2006) was calculated for each structural protein of each phage (Supplementary Table S1).

Data from the mass spectroscopy-quantified peptide abundances was used to model the behavior of the phage structural proteins run on an SDS-PAGE gel. Band width in the model gel was based on the NSAF values for each protein with detected peptides. Band vertical position was based on molecular weights predicted from amino acid sequences. The data were compiled for visualization in a custom R script^1,2^. For the largest band for each phage, 3-D predictions were modeled using I-TASSER (default settings) to test the hypothesis that they were phage major capsid proteins (Yang et al., 2015). The top-scoring model for each putative major capsid protein was further verified using ProSA to confirm that z-scores of all input structures were within the range typically found for native proteins of similar size (default settings; Wiederstein and Sippl, 2007). The Research Collaboratory for Structural Bioinformatics Protein Data Bank (RCSB PDB; Rose et al., 2017) was searched for the solved protein structure most similar to the structures predicted for the PSA phage major capsid proteins.

### Sequencing and Annotation

Phage genomes were sequenced on the Illumina HiSeq platform and the PSA-HS4 genome closed using Sanger sequencing. ORFs were predicted using prodigal (default parameters; Hyatt et al., 2010). Annotations were made based on a combination of structural proteomics, domain identification in Pfam database (e-value < 0.001; Finn et al., 2010), BLASTP-identified (Altschul et al., 1990) homology to sequences in National Center for Biotechnology Information’s (NCBI) non-redundant protein database (nr; January 2017), and tRNAs were searched for using tRNAscan-SE. Percentage of shared genes between the *Pseudoalteromonas* phages was based on an all-against-all comparison [BLASTP bit score > 75, per (Lavigne et al., 2008)]. Genome synteny plots were generated using Easyfig (v2.1; Sullivan et al., 2011) based on a full-genome BLASTN search, or occasionally TBLASTX where noted. The scope for whole-genome similarity to known phages was expanded beyond sequenced phage isolated to include prophages integrated in sequenced *Pseudoalteromonas* genomes. Prophages were predicted using VirSorter (version 1.0.2; Roux et al., 2015a) in RefSeq complete genomes and Whole Genome Sequencing (WGS) projects for organisms in the Order Alteromonadales, as well as sequenced Helgoland *Pseudoalteromonas* genomes (Duhaime et al., 2016). A heatmap was used to display relationships between *Pseudoalteromonas* phage and prophage genomes based on number of shared proteins. The orders of the genomes on the x- and y-axes were determined by the maximum distance method to encourage the self-hits and within-genus members to be attracted to the diagonal.

All predicted proteins from 43 global virus metagenomes, collectively the ‘*Tara* Oceans Viromes (TOV)’ dataset (Brum et al., 2015) were searched (blastp) against virus sequences in RefSeq (v70, May 2015). All TOV proteins with a bit score > 50 and e-value < 0.001 were considered already affiliated to existing known proteins. The remaining unaffiliated proteins were searched (blastp) against the 655 PSA phage proteins. Those with significant homology (bit > 50 and e-value < 1e-7) to PSA phage proteins were considered “newly affiliated.” PCs that did not contain already-existing sequences from RefSeq were identified as “newly annotated” as PSA phage protein homologs. If one or more member was similar to a PSA phage protein, the PC was deemed newly affiliated.

All raw data and code for data analysis and figure generation have been made publically available by webserver^3^ and github repository^4^.

## Results and Discussion

### Eight Helgoland *Pseudoalteromonas* Phages Constitute Five New Genera

**FIGURE 1:**
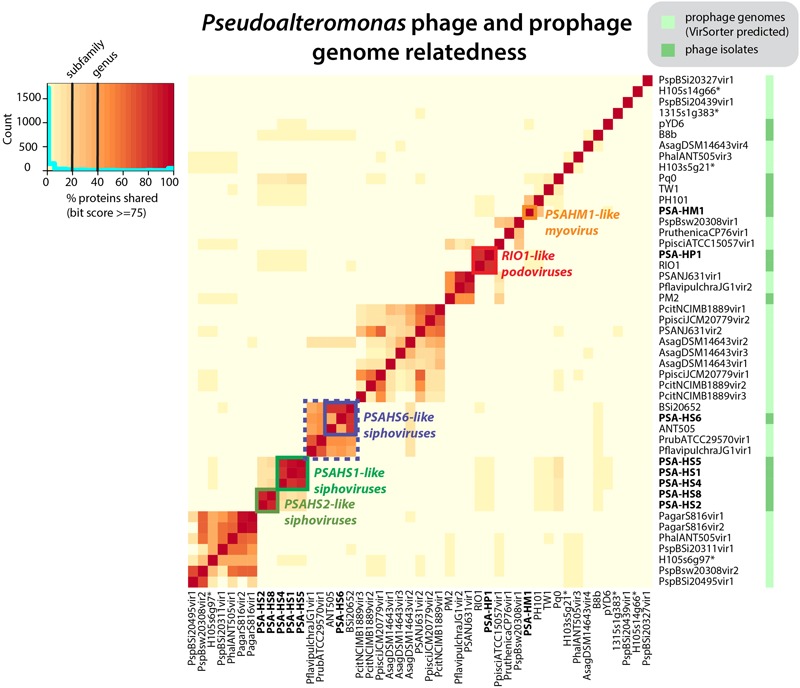
*Pseudoalteromonas* (PSA) phage genera, as determined by shared protein content between all sequenced PSA phages and prophages identified *in silico*. Phage isolates and prophages predicted from sequenced *Pseudoalteromonas* genomes are differentiated on the right hand color strip by dark and light green color blocks, respectively. Legend denotes subfamily (>20% proteins shared) and genus (40% proteins shared) delineations, per (Lavigne et al., 2008, 2009). Helgoland PSA phage is indicated by bold text. Candidate new genera comprised of sequenced Helgoland PSA phages are outlined in thick color blocks. ^∗^Indicates prophages identified in sequenced Helgoland *Pseudoalteromonas* spp. strains.

**FIGURE 2:**
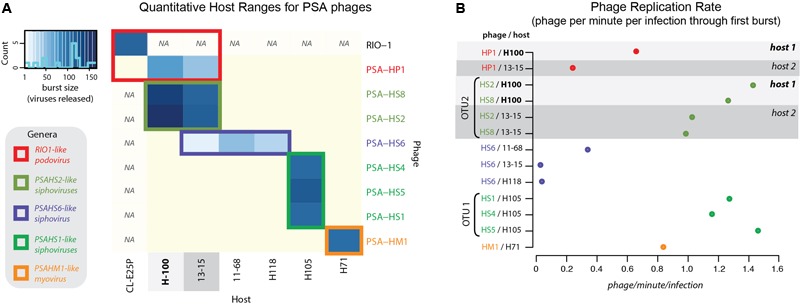
**(A)** Quantitative host range analyses, whereby shade of blue reflects the burst size of the phage in each infection. Non-blue boxes indicate that the host was challenged with the phage, but that no infection occurred. Boxes with “na” indicate that this infection was not tested. Count bar inset in the upper left shows the distribution of burst sizes across all virus–host pairs represented in the heatmap. **(B)** Virus production rates for Helgoland *Pseudoalteromonas* phages and hosts. Rates are calculated as number of phage produced per infection during the first step of the one-step virus production curve (Supplementary Figure S6). Light and dark gray bands emphasize like hosts when “phage OTU” infection traits are contrasted, as referenced later in conceptual model proposed in **Figure 7**.

The sequenced eight *Pseudoalteromonas* spp. phage genomes ranged in size from 35.3 to 129.4 kb and in G+C content from 35.7 to 44.7% (**Table 1**). Morphologically, the phages represented all three families in the order *Caudovirales*, with six siphoviruses, one podovirus, and one myovirus (**Figure 1**). These phages infect six closely related strains of *Pseudoalteromonas* sp. that varied by <2% 16S rRNA gene nucleotide identity (**Figure 2A** and Supplementary Figure S2).

**Table 1:** Overview of Helgoland *Pseudoalteromonas* phage genera features.

Family; proposed candidate genus	Phage	Genome size (bp)	G+C, %	ORF#	Phage isolation location	Genus features
Podoviridae; RIO1-like virus	PSA-HP1	45035	44.67	57	North Sea	Members of the VpV262-like cluster have VpV262-like structural module arrangement (ter-port-scaff-cap) that differs from T7 organization (Hardies et al., 2013). Lineages of VpV262-likes have dissimilar replicative modules, see incongruence TerL (structural) and DNApol (replication) phylogenies (Supplementary Figures S4, S5).
	*Pseudoalteromonas* phage RIO-1 (Hardies et al., 2013)	43882	44.7	56	East Sea, South Korea	
Myoviridae; PSAHM1-like virus	PSA-HM1	129401	35.73	225	North Sea	Shares 29% proteins and forms sub-family with PSA phage PH101 (**Figure 6**). Contains 27 T4-like genes (Supplementary Table S1); only one is structural. This is a novel non-T4-like myovirus genus.
Siphoviridae; PSAHS1-like virus	PSA-HS1	36769	40.52	62	North Sea	Likely capable integrating into host genomes; contain ERF required for homologous recombination and Cro/C1 transcriptional repressor used to maintain lysogeny.
	PSA-HS4ˆ (alias H105/1)	38739	40.38	68		
	PSA-HS5	37230	40.39	62		
Siphoviridae; PSAHS2-like virus	PSA-HS2	37728	40.21	63	North Sea	Lacks the canonical indicators of integration, e.g., in PSAHS1-likes
	PSA-HS8	37774	40.05	64		
Siphoviridae; PSAHS6-like virus	PSA-HS6	35328	40.21	54	North Sea	PSA-HS6 is isolated; others are prophages. Hybrids of sipho non-contractile tail fused to a myo Mu-like head. Fusion also seen in *Pseudomonas* phages DS3112-likes (**Figure 5B**). Mu backbone likely ancestral (Wang et al., 2003); tail formation modules more recent.
	*Pseudoalteromonas* BSi20652, ANT505, and CP76 prophages	na	na	na	Spanish salterns, Antarctica	

*ˆPSA-HS4 is resequenced and now complete genome of *Pseudoalteromonas* phage H105/1 (RefSeq NC_015293).*

The eight Helgoland *Pseudoalteromonas* phages belong to five new or candidate genera (delineated in **Figure 1**; described in **Table 1**) proposed here based on genome-based taxonomic guidelines (>40% proteins shared; Lavigne et al., 2008, 2009). Four of these Helgoland *Pseudoalteromonas* genera were novel, bringing the number of *Pseudoalteromonas* phage genera represented by isolated phages to a total of 11 (**Figure 1**). Seven of these are currently represented by a single phage. Only Helgoland podovirus PSA-HP1 formed a genus-level cluster with a previously sequenced phage isolate, *Pseudoalteromonas* phage RIO-1, which was isolated from the East Sea, South Korea, on 10 December 2007 (Hardies et al., 2013).

Phage genomes coincidentally sequenced during microbial host sequencing projects represent a recently illuminated source of phage genomic diversity (Roux et al., 2015b). As such, the 31 *Pseudoalteromonas* phages predicted to exist in sequenced *Pseudoalteromonas* genome projects (as prophages or extrachromosomal elements) were considered in this whole genome analysis (**Figure 1**; predicted prophages listed in Supplementary Table S2). When all *Pseudoalteromonas* phages and prophages are considered, 27 genera are resolved based on shared genome content, 18 of which are single phage or prophage genera (Supplementary Figure S1 and Table S1). The isolated *Pseudoalteromonas* phages and predicted *Pseudoalteromonas* prophages showed distinct clustering, which suggests minimal flow of genomic material between *Pseudoalteromonas* phages of these two contrasting lifestyles. Notably, the prophages include four from Helgoland *Pseudoalteromonas* hosts (strains H105, H103, and 13–15)—consistent with the dominant trend, none of these shared genome content with Helgoland phage isolates (**Figure 1**). The exceptions to this trend were Helgoland *Pseudomonas* phage PSA-HS6 and *Pseudomonas* phage PM2, two isolates that clustered in genera-level groups with predicted prophages (**Figure 1**). Both of these phages contain genes that suggest they may be capable of latent infections either through integration into their host genomes (PSA-HS6, discussed below) or as circularized plasmids (PM2; Männistö et al., 1999).

Of the 656 new *Pseudoalteromonas* phage proteins discovered in the Helgoland phage genomes, 285 were more similar to proteins in a global collection of ocean viral metagenomes—the combined ‘POV’ (Hurwitz and Sullivan, 2013) and ‘TOV’ (Brum et al., 2015) datasets—than to proteins in NCBI’s RefSeq database. Of the sequences comprising existing PCs generated from POV and TOV (Hurwitz and Sullivan, 2013; Brum et al., 2015), 76 were homologous to the new *Pseudoalteromonas* phage proteins. This association informed 11 existing PCs that had been unannotated previously as bonafide phage clusters (Supplementary Table S3). Further, the PSA phage proteins were homologous to 422 singleton proteins that previously had not belonged to a virus PC, thereby forming new ocean virus PCs and shedding valuable light on a component of unknown virus sequence space.

### Identification of Phage Structural Proteomes

As structural proteins in new phages can rarely be annotated by sequence homology alone (Brum et al., 2016b), we chose to experimentally identify the proteins in purified viral particles using mass spectroscopy-based shotgun proteomics. One phage from each genus and two for the *PSAHS1-likes* were used to identify and annotate 94 proteins associated with the phage virions. Of these proteins, 49 were not previously known to be structural. Ten of the confirmed structural proteins belong to protein families with domains of unknown function (Pfam ‘DUFs’ 935, 1320, 4128, 4055, 3383, 2612; Supplementary Table S3), which help to propagate the structural annotations to a total of 840 DUF sequences in Pfam (counts as of January 2017). All but two of these DUF-containing structural proteins (PSAHS1_00032 and PSAHS4_00014) affiliated with existing global ocean viral PCs (Supplementary Table S3). As they were found in the predominantly non-ocean phages and prophages in public sequence databases as well as in ocean viromes, these data indicated that the core structural functions of PSA phages are conserved across diverse habitats.

**FIGURE 3:**
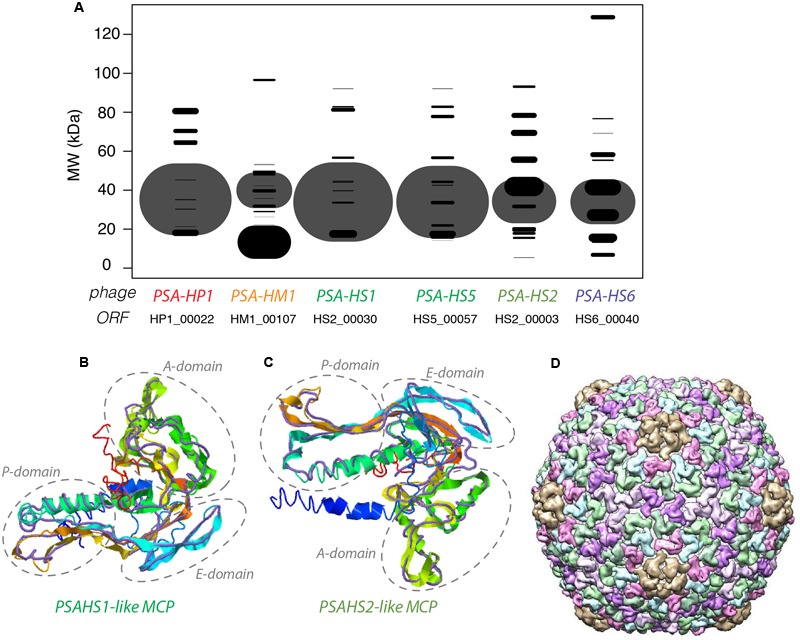
**(A)** Modeled 2D SDS-PAGE gel representing structural proteins of a type phage from each of the new Helgoland *Pseudoalteromonas* phage genera. Band width is based on mass spectroscopy-quantified peptide abundances, as calculated by the normalized spectral abundance factor (NSAF; 2006)—essentially the predicted relative abundance of each protein in the virion of each phage. The size of the “fragment” represented the per phage NSAF. The largest bands in each row (phage major capsid proteins) are shown with transparency to allow visualization of overlapping bands (all other black bands). Band vertical position is based on molecular weights predicted from amino acid sequences. **(B)** 3-D prediction of PSA-HS1 phage major capsid protein (HS1_00030) with cryo-EM resolved backbone (purple) of *Bacillus subtilis* bacteriophage SPP1 protein, gp13. **(C)** 3-D prediction of PSA-HS8 phage major capsid protein (HS8_00019) with cryo-EM resolved backbone (purple) of *Bacillus subtilis* bacteriophage SPP1 protein, gp13. **(D)** Reconstructed capsid of *Bacillus subtilis* bacteriophage SPP1 protein, comprised of multiple gp13 subunits, which we predict to be analogous to the PSA-HS1- and PSA-HS2-like genera capsids.

The NSAF (Paoletti et al., 2006) was calculated for each proteome to quantitatively assess the relative abundances of the structural proteins comprising each phage. The proteins with the most abundant peptides in PSA-HP1, PSA-HM1, and PSA-HS6 were annotated as major capsid proteins or major capsid subunits based on their high peptide coverage (**Figure 3A**), as well as significant amino acid sequence homology with phage proteins in GenBank (Supplementary Table S3). Among the *PSAHS1-like* and *PSAHS2-like* siphoviruses, each representative proteome contained a single protein whose peptide coverage numerically dwarfed the others by 2- to 10-fold (**Figure 3A**). Based on the trend seen in the other PSA phages and the knowledge that major capsid proteins can constitute 57–65% of siphovirus proteomes (Buchwald et al., 1970; Zweig and Cummings, 1973) and are commonly the most abundant protein of other phage families (Simoliūnas et al., 2013), we hypothesized that the high-abundance proteins and their homologs in the five *PSAHS1-like* and *PSAHS2-like* siphoviruses were major capsid proteins. This hypothesis was confirmed by the generation of 3D protein structure predictions of the putative PSA phage major capsid proteins followed by a comparison of these models with known structures in RCSB PDB (Rose et al., 2017). The z-scores of all predicted PSA major capsid protein structures were within the range of scores typically found for native proteins of similar size (Supplementary Figure S3; Wiederstein and Sippl, 2007), which provided confidence in the evidence based on structure prediction comparisons. For both the *PSAHS1-like* and *PSAHS2-like* Helgoland PSA siphoviruses, the best-aligned structure was a cryo-EM resolved protein, gp13, from *Bacillus subtilis* bacteriophage SPP1 (**Figures 3B–D**; White et al., 2012). The assembled capsid of phage SPP1 is comprised of multiple gp13 hexamers that anchor a capsid spike protein, gp12 (White et al., 2012). Based on these results, we predict the capsid structure of the *PSAHS1-* and *PSAHS2-like* genera phages to be analogous to the SPP1 phage capsid (**Figure 3D**). These analyses enabled the annotation of previously unknown proteins as major capsid proteins in the five new Helgoland PSA siphoviruses.

**FIGURE 4:**
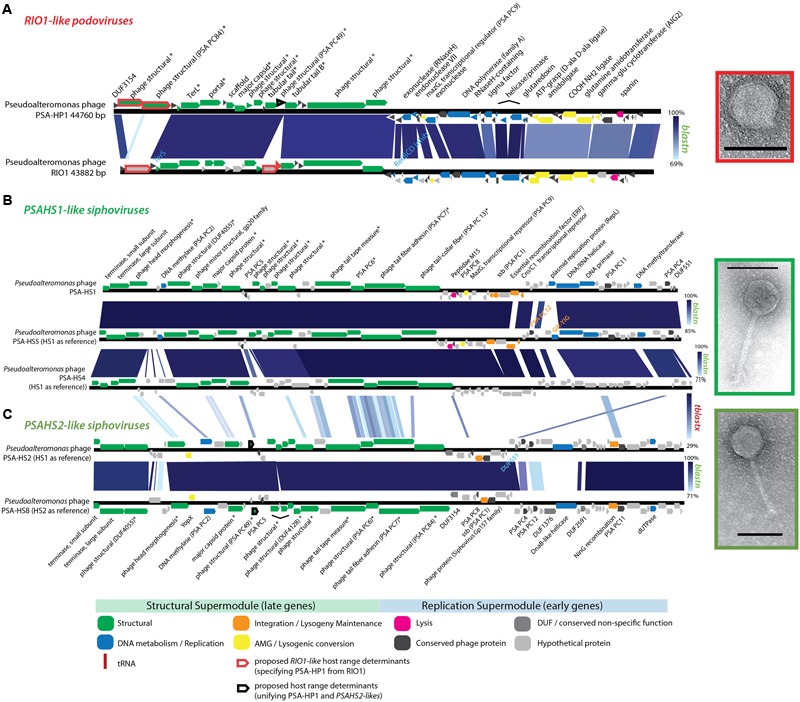
**(A)** Full-genome comparison of PSA-HP1 and *Pseudoalteromonas* phage RIO-1 (East Sea, South Korea; Hardies et al., 2013) based on BLASTN similarity. Red outline indicates the five structural proteins not shared between PSA-HP1 and RIO1 that are implicated in their host range determination. Black outline indicates genes shared with RIO1-like phages and *PSAHS2-like* phages, but not with the *PSAHS1-like* phages. **(B)** Full-genome comparison of the three Helgoland phages belonging to the *PSAHS1-like* phage genus based on BLASTN similarity. **(C)** Full-genome comparison of the two Helgoland phages belonging to the *PSAHS2-like* phage genus based on BLASTN similarity. Connections between **(B,C)** indicate similarity between the *PSAHS1-like* and *PSAHS2-like* genera based on TBLASTX analysis. ^∗^ Denotes proteins confirmed as structural by mass spectroscopy-based proteomics.

Structural proteomics improved the resolution of structural module localization and, when combined with host range analysis, served to implicate three structural proteins as host range determinants. The *RIO-likes* (PSA-HP1 and RIO-1), while highly syntenic, do not share five proteins in their structural modules that may be involved in host specificity (red outlined ORFs, **Figure 4A**). In our host range analyses, PSA-HP1 did not infect *Pseudoalteromonas marina* str. CL-E25P (**Figure 2**), the strain used to isolate RIO-1. We hypothesize that the proteins not shared between these phages may be involved in host range determination. Contrastingly, podovirus PSA-HP1 and the *PSAHS2-like* siphoviruses have identical host ranges (**Figure 2**) and their only genotypic similarities are two structural proteins (PC49 and PC84, **Table 2**). Of these, one protein (PSA-HP1 ORF 17, PC49; black outlined ORFs, **Figure 4A**) is not found in the *PSAHS1-like* siphoviruses, which do *not* overlap in host range with PSA-HP1. We hypothesize that PC49 may play a role in host range and thereby bridging these *Pseudoalteromonas* phage families. We propose these candidates for future protein–protein interaction studies.

**Table 2:** Overview of proteins shared within and between genera.

Family		Podo	Myo	Sipho		
	*Genus*	*RIO1-like*	*HM1-like*	*HS1-like*	*HS2-like*	*HS6-like*
**Podo**	***RIO1-like***	
**Myo**	***HM1-like***	None	
**Sipho**	***HS1-like***	MazG nucleotide pyrophosphohydrolase (PSA PC9)	
	***HS2-like***	Phage structural protein (PSA PC49) Phage structural protein (PSA PC84)	DNA methylase (PSA PC2)	Phage structural protein	
				DNA methylase (PSA PC2)	
				Phage head morphogenesis protein, SPP1 gp7 family	
				Phage protein	
				Phage protein (PSA PC11)	
				Phage protein (PSA PC4)	
				Phage tail fiber adhesin (PSA PC7)	
				Phage structural protein (PSA PC6)	
				Phage protein (PSA PC8)	
				Phage structural protein (PSA PC84)	
				Phage protein (PSA PC5)	
				Phage tail tape measure protein	
	***HS6-like***	None	None	None	None	

*Proteins shared between Helgoland *Pseudoalteromonas* phage groups with greater than or equal to 75 blast bit score or 40% amino acid identity, per (Lavigne et al., 2008, 2009).*

### A Sipho-Myo Hybrid with Mu-Type Replication: A Rarely Described Feature of the Ocean Virus Landscape

**FIGURE 5:**
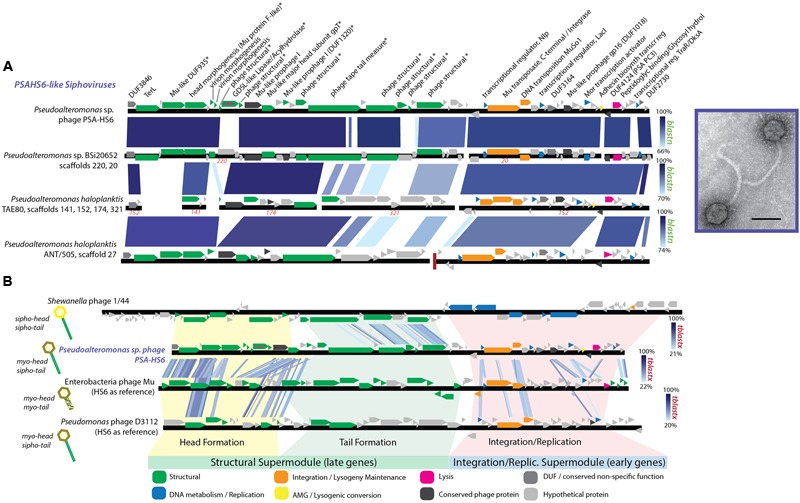
**(A)** Full-genome comparison based on BLASTN similarity between PSA-HS6 and three integrated prophages identified in sequenced *Pseudoalteromonas* host genomes in the Whole Genome Shotgun (WGS) dataset: *Pseudoalteromonas* sp. BSi20652 contigs 20 (BADT01000020) and 220 (BADT01000220); *Pseudoalteromonas haloplanktis* TAE80 contigs 141 (AUTM01000141), 321 (AUTM01000321), 174 (AUTM01000174), and 152 (AUTM01000152); *Pseudoalteromonas haloplanktis* ANT/505 contig 27 (NZ_ADOP01000027). ^∗^ Denotes proteins confirmed as structural by mass spectroscopy-based proteomics. **(B)** Full-genome comparison based on TBLASTX similarity between PSA-HS6, Enterobacteria phage Mu, *Pseudomonas* phage D3112, and *Shewanella* phage 1/44. This representation shows the conserved elements in the head and integration/replication modules shared between PSA-HS6 and Enterobacteria phage Mu, as well as *Pseudomonas* phage D3112—representing a group of phage where this Mu head and siphovirus tail has been previously described (Wang et al., 2003; Cazares et al., 2014). Further, this representation depicts the siphovirus-like tail shared between PSA-HS6 and *Shewanella* phage 1/44.

The proposed *PSAHS6-like* genus contains siphovirus PSA-HS6 as its sole isolated member, but shares 90–92.4% ANI and 66–87% of its proteins with three prophages integrated in sequenced *Pseudoalteromonas* genomes in GenBank’s Whole Genome Shotgun (WGS) database (**Figures 1, 5A**). The *PSAHS6-like* phages share similarity with neither the *PSAHS1-like* nor *PSAHS2-like* Helgoland siphoviruses nor any other Helgoland phages (**Figure 1**). Rather, morphology and protein homology suggest the *PSAHS6-like* phages are modular hybrids composed of a siphovirus-like non-contractile tail fused to a myovirus Mu-like head (**Figure 5B**). Protein homology suggested the *PSAHS6-like* phages replicate by Mu-type transposition, which implies obligate integration into their host genomes and the capacity to remain as prophages in a host lysogen (Toussaint et al., 1994). The *PSAHS6-like* genomes contain numerous Mu-like conserved proteins localized in the two canonical Mu regions (Morgan et al., 2002): (i) head formation among the late genes and (ii) transposition and replication functions among the early genes (**Figure 5A**). Contrastingly, the *PSAHS6-like* tail formation module is homologous to non-Mu siphoviruses (e.g., *Shewanella* phage 1/44; **Figure 5B**). The similarity to Mu-like phages, supported by the location of the PSA-HS6 large terminase within the “Mu-like headful packaging” terminases (Supplementary Figure S5) and their propensity to be found integrated in host genomes, point to *PSAHS6-like* phages replicating via Mu-like transposition. Considering the propensity for Mu-like phages to package host DNA during transposition and replication (Toussaint et al., 1994) and that the *PSAHS6-like* superfamily is the second largest superfamily of *Pseudoalteromonas* phages (dotted box **Figure 1**), *PSAHS6-like* phages may be important components of the *Pseudoalteromonas* mobilome and sources of diversification and genomic exchange within this genus.

While similar Mu head-siphovirus tail fusions have been reported in a global collection of 12 phages and prophages of clinical *Pseudomonas aeruginosa* isolates (Wang et al., 2003; Cazares et al., 2014), descriptions of Mu-type sipho-myo hybrids isolated from the marine environment are less common. Integrated Mu-like prophages are thought to be not inducible by mitomycin C (Paul, 2008)—though exceptions exist (Zheng et al., 2014). As such, quantifying their abundance in induced metagenome studies (e.g., McDaniel et al., 2008; Brum et al., 2016a) will be challenging. However, with phage and host in culture, we can now explore the environmental triggers that drive this Mu-type replicating virus to oscillate between lytic and lysogenic replication to better understand a class of lysogeny not yet explored in the environment.

### Host Takeover Mechanisms Inferred from *Pseudoalteromonas* Phage Genomes

#### Hijacking Host Stress Response: MazG, DksA, PhoH

In order to respond to rapid environmental changes and resource availability, bacteria often adjust their global regulatory networks at the level of transcription (reviewed in Shimizu, 2013). One such mechanism is through the rapid production of a stringent response effector nucleotide, guanosine 3′,5′-bispyrophosphate (ppGpp), in response to myriad nutritional stresses (Barker et al., 2001; Gross et al., 2006). ppGpp directly binds to promoter regions of RNA polymerase (RNAP) and globally influences transcription (Barker et al., 2001; Gross et al., 2006). Phages appear to have evolved mechanisms to tweak ppGpp-mediated response of host cells to the environment (Borbély et al., 1980; Clokie and Mann, 2006; Bryan et al., 2008).

MazG is a protein known to stall or reverse starvation-induced programmed cell death in *Escherichia coli* by decreasing the cellular pool of ppGpp (Gross et al., 2006). MazG, found in half the genomes presented here (podovirus PSA-HP1, the three *PSAHS1-like* siphoviruses; **Figure 4B** and Supplementary Table S3), is over-represented in marine phages (Duhaime et al., 2011) and shared among all known T4-like cyanophages (Sullivan et al., 2010). When carried by phage and expressed during infection, MazG is thought to impede the global dampening of metabolic processes, thereby ensuring prolonged phage replication in a starving host (Clokie and Mann, 2006; Bryan et al., 2008). Further studies are needed to resolve the role of MazG in infection dynamics, particularly under conditions of nutrient-limitation induced host stress.

DksA is a critical component of the rRNA transcription initiation machinery that binds to RNA polymerase and also influences the regulation of rRNA promoters by ppGpp (Paul et al., 2004). The Helgoland *Pseudoalteromonas* Mu-like siphovirus, PSA-HS6, and its relatives have DksA family transcriptional regulators (**Figure 5A** and Supplementary Table S3). We hypothesize that this system may provide a mechanism by which phage activity is modulated based on their host’s physiological status, e.g., in the switch between lytic and lysogenic lifestyles.

**FIGURE 6:**
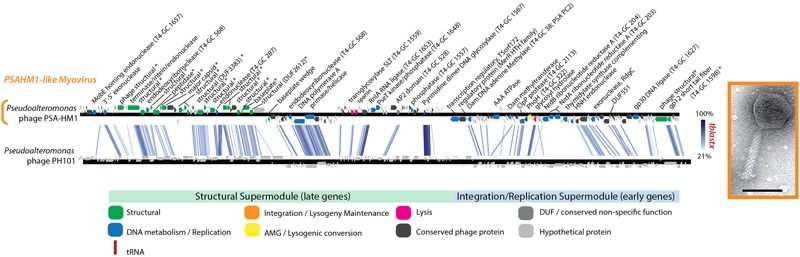
Full-genome comparison based on TBLASTX similarity between PSA-HM1 and *Pseudoalteromonas* phage PH101. These phages do not belong to the same genera, based on genome similarity criteria. ^∗^ Denotes proteins confirmed as structural by mass spectroscopy-based proteomics.

PhoH is another protein commonly found in phage genomes. *phoH* is a core gene shared by 16 marine T4-like cyanophages compared in one study (Sullivan et al., 2010) and is found in other marine viruses (Goldsmith et al., 2011), including non-cyanobacterial myovirus Vibriophage KVP40 (Miller et al., 2003), podovirus Roseophage SIO1 (Rohwer et al., 2000), and the Helgoland myovirus PSA-HM1 presented here (**Figure 6** and Supplementary Table S3). This presence across diverse marine host types, a pattern that PSA-HM1 strengthens, hints toward *phoH* conferring a benefit to marine phages infecting ocean-dwelling hosts. PhoH belongs to the phosphate (pho) regulon, whose transcription is inducible under phosphate limitation (Kim et al., 1993). However, the specific dynamics of PhoH regulation are not uniform across systems (Goldsmith et al., 2011). For instance, during phosphate starvation the expression of the *phoH* gene increases in *Escherichia coli* (Wanner, 1993) and *Corynebacterium glutamicum* (Ishige et al., 2003), decreases in marine *Synechococcus* (Tetu et al., 2009), and is unchanged in *Prochlorococcus* MED4 and 9313 (Martiny et al., 2006). In the absence of explicit phosphate-limitation in growth media, *phoH* is upregulated in *Prochlorococcus* MED4 during the late stages of infection by podovirus P-SSP7 (Lindell et al., 2007). We speculate that this may be due to cellular stress due to P-limitation as a result of the production of new virions that require more P (relative to C and N) than the ratio required by uninfected host cells (Jover et al., 2014). PhoH, believed to be a cytoplasmic protein involved in the uptake of phosphate under conditions of phosphate starvation (Kim et al., 1993; Makino et al., 1994), may confer an advantage to phages that carry it by supplementing phosphate uptake during the onset of P-limitation in the late stages of infection. Phosphorus indeed limits productivity in many ocean habitats and has been implicated as a dominant selective force in shaping microbial population heterogeneity (Coleman and Chisholm, 2010). Clearly, further experimental studies of infection under phosphate limitation are needed resolve these hypotheses and the role of phage—carried PhoH during infection.

#### Peptidoglycan Modification Cassette

The *RIO1-like* podoviruses carry a seven-gene cassette of peptidoglycan modification genes (PSA-HP1 ORFs 40–47; **Figure 4** and Supplementary Table S3) hypothesized to play a role in altering the host cell surface after infection to prevent superinfection by additional phages (Iyer et al., 2002). Similar genes have been found in other podoviruses (Hardies et al., 2013). Collectively these genes encode proteins that perform functions necessary to synthesize three unusual linkages in peptidoglycan peptide side chains (Iyer et al., 2002; Hardies et al., 2013), including a gamma-glutamyl amidoligase (PSAHP1_00043), a second uncharacterized amidoligase (PSAHP1_00046), and an ATP grasp enzyme (PSAHP1_00047). Beyond synthesis, there are also peptidoglycan degradation genes encoded in the PSA-HP1 genome, including gamma-glutamyl cyclotransferase (PSAHP1_00040) and glutamine amidotransferase (PSAHP1_00042). This pathway is conserved across several marine and non-marine podovirus genomes, including *P*s*eudoalteromonas* phage RIO-1, Enterophage phiEco32, *Salmonella* phage 7-11, *Pseudomonas* phages PA11, tf, MR299-2, LUZ24, and PaP3, and *Salinivibrio* phage CW02 (Hardies et al., 2013). In these genomes, the host material recycling genes are located amidst genes involved in transcription, DNA metabolism, and replication – a pattern observed in other podoviruses (Lindell et al., 2007; Gao et al., 2012). Notably, genes in this region are co-transcribed in *Prochlorococcus* phage P-SSP7 replication (Lindell et al., 2007), suggesting that this shared genome organization could facilitate efficient recycling of limiting cellular resources during infection.

### Advancing the Phage OTU Concept: Fitness-Determining Traits Are Conserved within Genome Groups of 95% ANI

**FIGURE 7:**
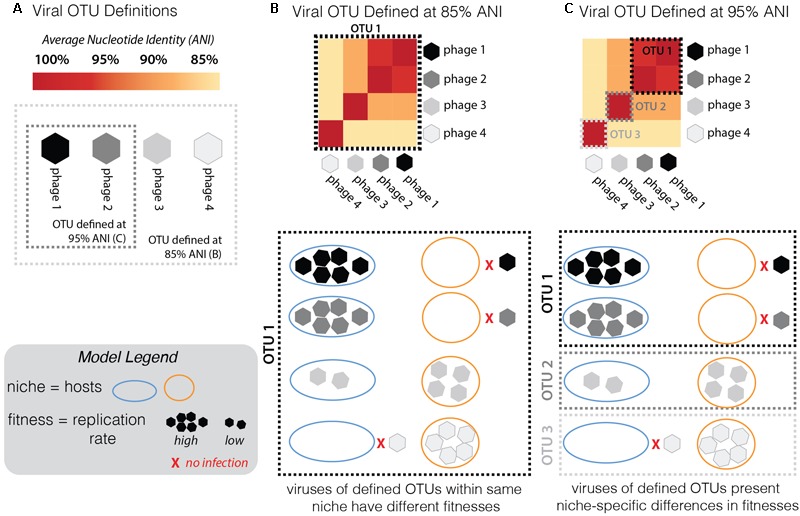
Conceptual model of the framework used here to assess the validity of the current working definition of a phage population, or “phage OTU.” **(A)** For a group of phages along a continuum of genome similarity (measured by average nucleotide identity, ANI) from 85 to 95%, we contrast the scenario whereby the phage OTU is defined at 85% (light gray dashed box) with the scenario when it is defined at 95% ANI (dark gray dashed box). To test the validity of these arbitrary groupings in establishing an effective definition for a phage OTU, one must consider fitness differences (measured by replication rates; see Model Legend) in different niches (hosts: “host 1” depicted by blue outline, “host 2” by orange; inability to infect is depicted by red “x”; see Model Legend). **(B)** Given the infection outcomes of the phages in this OTU 1 (defined by 85% ANI), there is no ecological cohesiveness. Phages in this OTU have different fitness outcomes (ranging from no infection to high reproduction rate on both hosts). **(C)** By redefining the phage OTU at 95% ANI, this same group of phages now falls into three distinct OTUs. Now the same infection traits observed by the distinct OTUs fall into ecologically cohesive patterns. Within each OTU, phages infect the same hosts with similar efficiencies.

An effective phage OTU definition should capture evolutionarily and ecologically cohesive populations (**Figure 7**). In other words, genotypes in a population at the defined OTU should display no fitness differences in the same niche space. This would result in a strong correlation between genome similarity, fitness, and niche space—indicative of ecological differentiation, as demonstrated in microbial populations (Polz et al., 2006; Cordero and Polz, 2014). To test this trait-based OTU-defining framework here, sensitive *Pseudoalteromonas* hosts were considered viral niches. Sensitivity of a host to a virus is not fully described by a binary relationship (e.g., infects/does not infect), but rather is a system-specific equation of, e.g., adsorption kinetics and infection mode (lytic/lysogenic decision), as demonstrated in marine *Cellulophaga* and its phages (Holmfeldt et al., 2014). Here, infection properties (burst sizes, latent periods, and replication rates) were tracked as traits indicative of viral fitness in the niche space tested (**Figure 7**). Notably, all infections were performed under identical media and culturing conditions. Theoretically, the full niche space of each virus would include the entire range of existing sensitive hosts, however, only part of this niche space could be explored here, as we were limited by the bacterial isolates available.

Previous studies have invoked a >95% ANI threshold to define and study the global biogeography of virus populations (Brum and Sullivan, 2015; Roux et al., 2016). In cyanophages, population genetic analyses support the validity of this cut-off in delineating populations under different selective pressures and where barriers to cross-population gene flow exist (Gregory et al., 2016). Others have shown the existence of stable cyanophage ecotypes that persist for decades and that exhibit distinct temporal and spatial patterns of abundance—though in this study the clusters were defined at >93 and >98% ANI for their defined “clusters” and “subclusters,” respectively (Marston and Martiny, 2016). Yet, the appropriateness of the genome-similarity-based threshold remains unexplored in non-cyanophage systems and has not yet included consideration of fitness-determining infection traits. We hypothesized that if this OTU definition were meaningful in the *Pseudoalteromonas* phage–host system, trait-based differentiation would arise consistent with the 95% ANI genome-similarity boundaries used currently to define phage populations (Brum et al., 2015; Gregory et al., 2016).

Two multi-phage OTUs emerged from the Helgoland PSA phage collection. The siphoviruses in the *PSAHS1-like* genus (PSA-HS4 and PSA-HS5) share 97.6–99% genome-wide ANI (**Figure 4B**) and those in the *PSAHS2-like* genus (PSA-HS2 and PSA-HS8) share 97.9% genome-wide ANI (**Figure 4C**). With this level of similarity, each set meet our criteria to be grouped into a single phage OTU (as in “OTU 1” in **Figure 7C**). The phenotypic trait data generated here for the multiple members of each phage OTU (e.g., burst sizes, host ranges, infection efficiencies; **Figure 2** and Supplementary Figure S2) suggest that the >95% ANI delineation represents meaningful ecological distinctions in this system. Specifically, when phages within each OTU have overlapping host ranges (i.e., occupy the same part of their niche), such as when HS2 and HS8 infect H100 (“host 1” in **Figure 2B**), they also have similar infection traits (burst sizes, latent periods, replication rates; **Figure 2**). However, this did not preclude different fitnesses when phages of an OTU occupied a different part of their niche, e.g., lower replication rates when phages of the *PSAHS2-like* OTU (PSA-HS2 and PSA-HS8) infected host 13–15 (“host 2”) instead of H100 (“host 1”; **Figure 2**). Phage fitness remained similar when phages of the same OTU occupied the same niche space (**Figure 2B**). These patterns provide evidence for niche-specific fitness-determining trait conservation within defined phage OTU populations.

On the contrary, while the PSA-HP1 and the observed niche space of the *PSAHS2-like* OTU population completely overlapped (i.e., infected the same hosts: H100 and 13-15; **Figure 2**), their fitnesses (i.e., phage replication rates) in this niche differ markedly (**Figure 7C**). In the niche space tested, these OTUs result in a distinct ecological impact. As the PSA-HP1 and the *PSAHS2-like* OTUs fail the genome-similarity criterion—as expected, considering their contrasting morphologies and thus structural gene sequences, they further represent “evolutionarily distinct” units.

While sample size is low and the full niche space of this set of OTUs has not been explored, these data and conceptual model suggest currently proposed genome-similarity thresholds delineate ecologically differentiated phage OTUs. Determination of fitness-conferring infection traits for the large collections of closely related phages (e.g., Pope et al., 2015; Gregory et al., 2016; Marston and Martiny, 2016) is a valuable metric that—while labor intensive to collect—would further test this OTU-defining model and enable the application of theory to advance viral community ecology.

## Conclusion

The analysis of these eight new Helgoland *Pseudoalteromonas* phages is a first step in the development of a model system that will continue to improve our understanding of viruses infecting particle-associated ocean copiotrophs. Future work can build upon the comparative genomic foundation of this *Pseudoalteromonas* model system to investigate (i) the impact of boom–bust dynamics on the evolution and ecology of viruses of copiotrophs, including implications for population genetics of both viruses and hosts, (ii) the adaptive mechanisms viruses have acquired to deal with their hosts’ responses to nutrient fluctuations in the environment, and (iii) the impacts of patchy infections in nature on both genome evolution and infection ecology. Beyond fundamental comparative genomic observations, exploration of genotype and phenotype linkages in the Helgoland *Pseudoalteromonas* phages offered insights into variability in infection traits within a genome-defined phage population, e.g., phage OTU. These data support a framework for future efforts of larger scale to evaluate the proposed phage OTU definition—a necessary building block in the pursuit to quantify the relative import of processes governing phage community ecology. Together with advances in virus–host interaction theory (Weitz et al., 2013), ecosystem models (Weitz et al., 2015), and approaches to integrate microbial omics and biogeochemical data at a global scale (Guidi et al., 2016), this new PSA phage–host model system provides insights into particle-associated virus–host interactions and brings us one step closer to developing a predictive understanding of how viruses alter natural ecosystems.

## Author Contributions

MD designed the experiments, performed analyses, and wrote the manuscript. NS performed laboratory experiments; acquired and analyzed infection data and critically edited the manuscript. SR performed bioinformatic analyses against POV and TOV and critically edited the manuscript. NV performed proteomic analyses. AW conceived experiments and critically edited the manuscript. MS designed experiments, critically edited the manuscript.

## Conflict of Interest Statement

The authors declare that the research was conducted in the absence of any commercial or financial relationships that could be construed as a potential conflict of interest.

## Footnotes

1 http://www-personal.umich.edu/~duhaimem/Rpubs_code/Helgoland_PSA_phage_Rpub.html

2 https://github.com/ecogenomix/PSAPhageGnms

3 http://www-personal.umich.edu/~duhaimem/Rpubs_code/Helgoland_PSA_phage_Rpub.html

4 https://github.com/ecogenomix/PSAPhageGnms
