# Supplementary material for: Comparative Omics and Trait Analyses of Marine Pseudoalteromonas Phages Advance the Phage OTU Concept
Source: Front Microbiol. 2017 Jul 6;8:1241. doi: 10.3389/fmicb.2017.01241 (PMC5498523; doi:10.3389/fmicb.2017.01241)
Supplement: Supplementary file 1 [file Data_Sheet_1.pdf]

Supplementary Material

Comparative omics and trait analyses of marine *Pseudoalteromonas* phages advance the phage OTU concept

Melissa B. Duhaime, Natalie Solonenko, Simon Roux, Nathan C. Verberkmoes, Antje Wichels[5], Matthew B. Sullivan

*** Correspondence:**
Melissa B. Duhaime: duhaimem@umich.edu, Matthew B. Sullivan mbsulli@gmail.com

1 Supplementary Figures and Tables

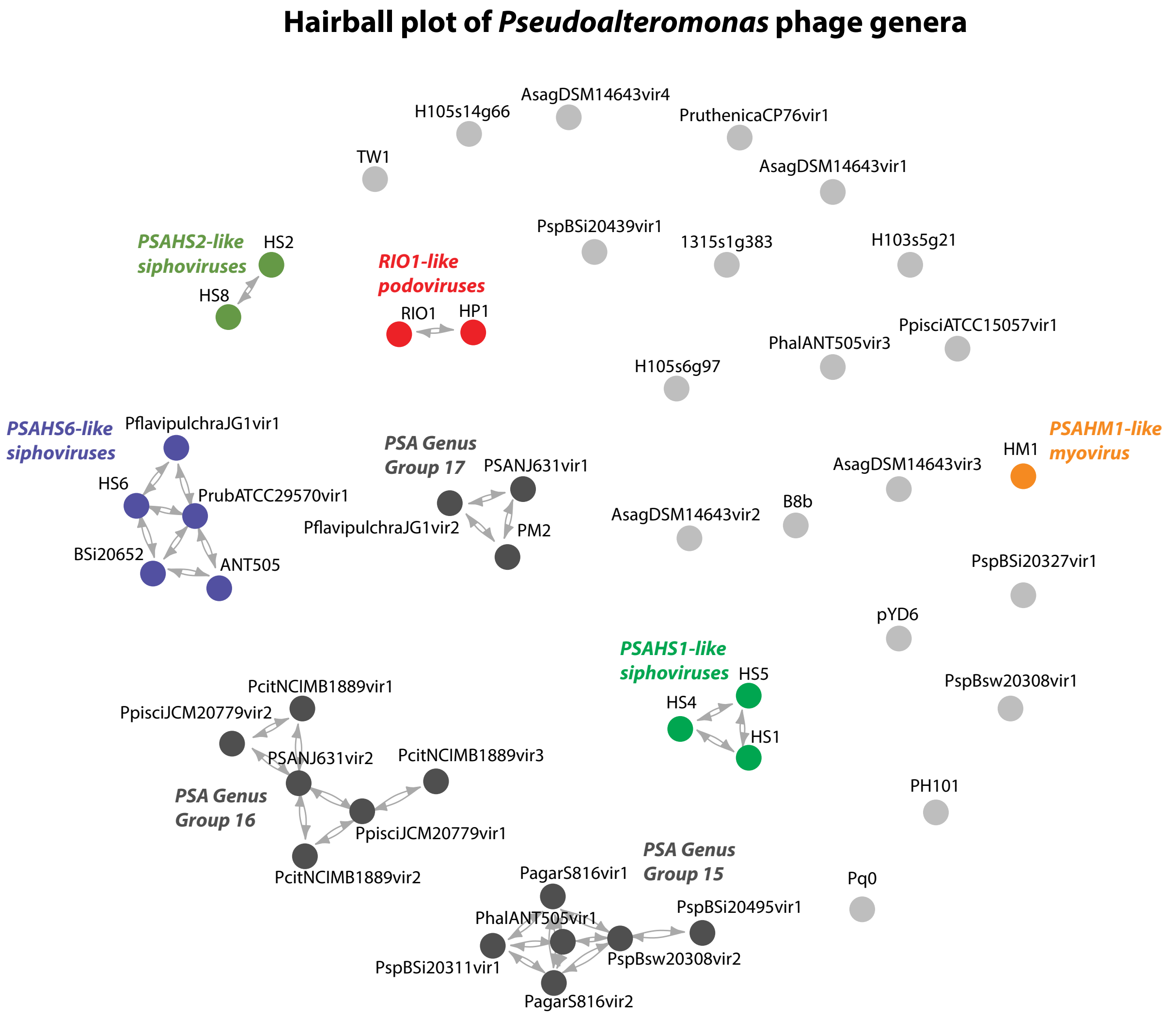

Supplementary Figure 1. Hairplot representation of the Pseudoalteromonas phage clusters. Directional arrows indicate that a phage shares 40% of its proteins with the phage it is pointing to. PSA Genera Group numbers are listed in Supplementary Table 1.

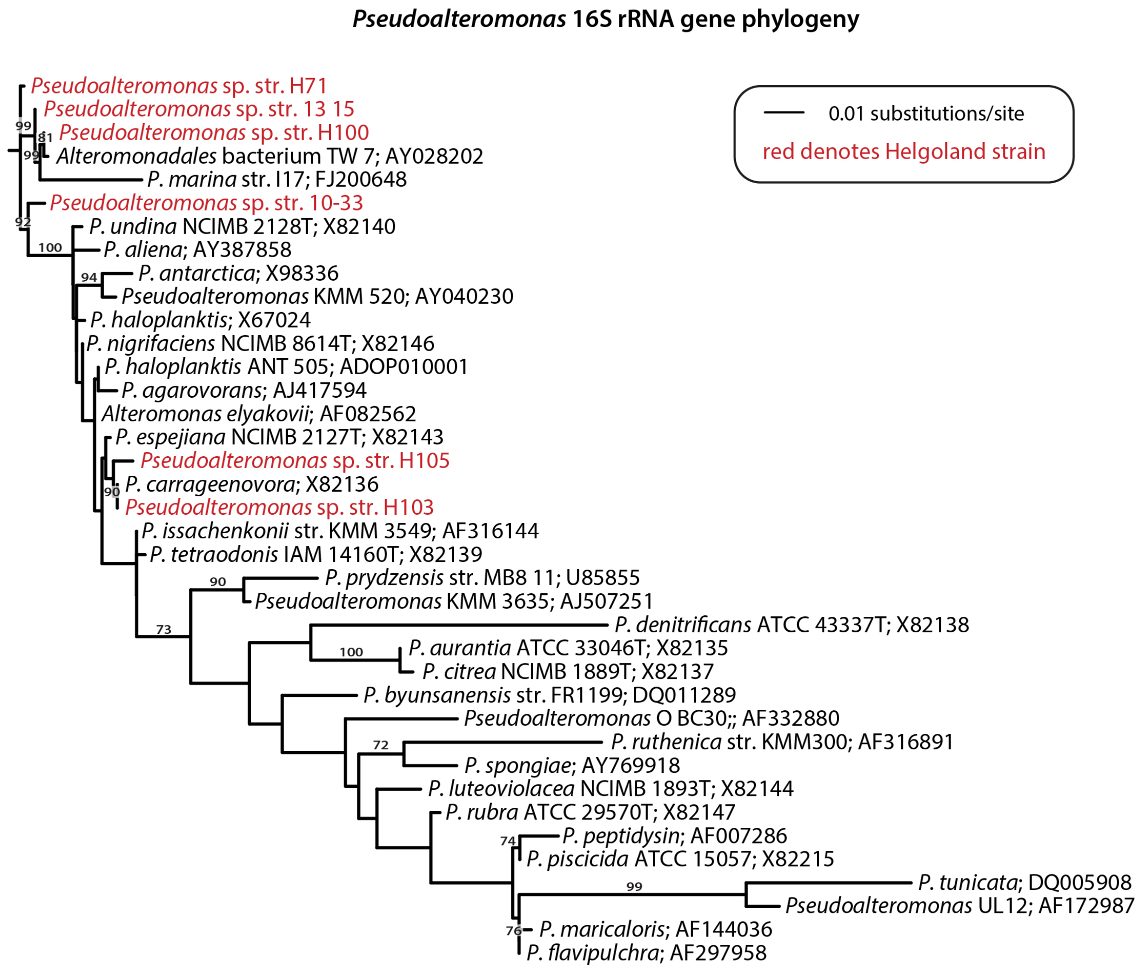

Supplementary Figure 2. 16S rRNA gene phylogeny for a subset of publicly available *Pseudoalteromonas* spp. and 6 Helgoland *Pseudoalteromonas* spp. (in red).

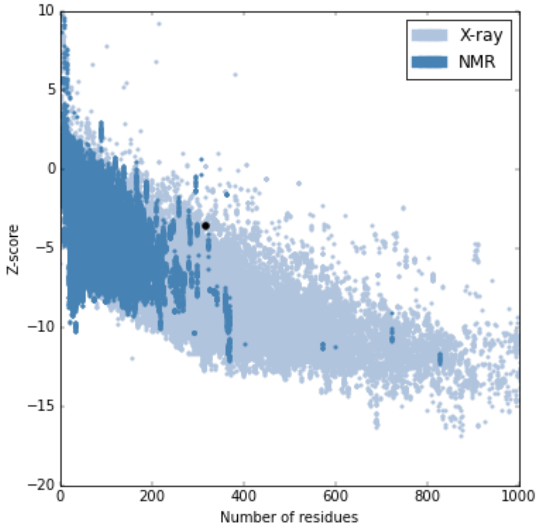

(A)

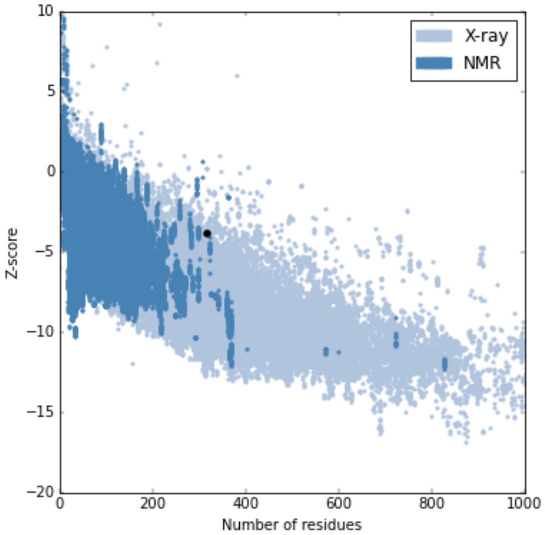

(B)

Supplementary Figure 3. Evaluation by ProSA software (Wiederstein and Sippl, 2007) of major capsid protein 3D model predicted by I-TASSER for: **(A)** PSAHS1_00030 and PSAHS5_00057, **(B)** PSAHS8_00019. All z-scores of predicted structures (black dot in panels A and B) fall in range of those expected based on empirical X-ray- and NMR-generated structural data.

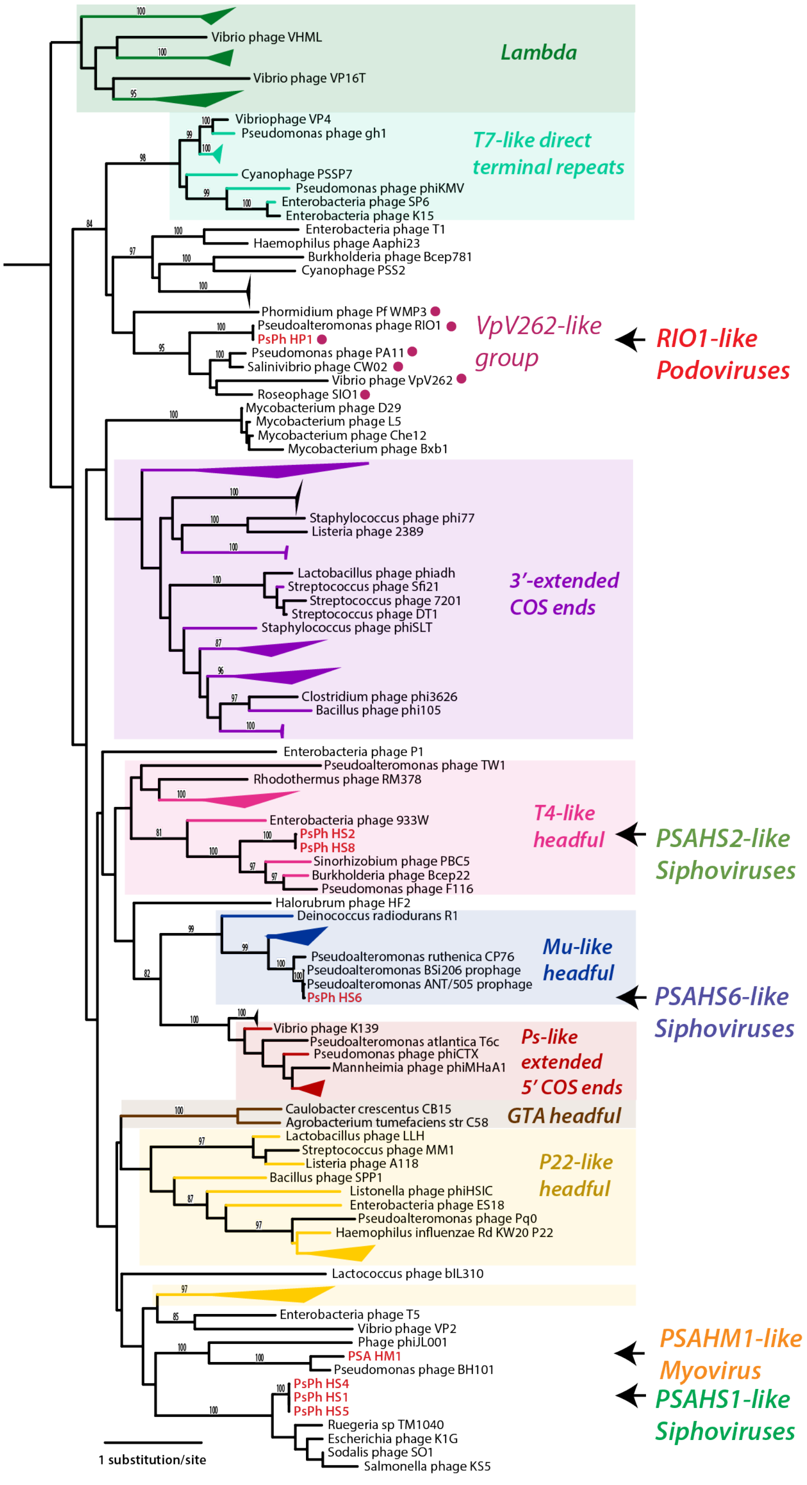

Supplementary Figure 4. Large terminase (terL) maximum likelihood-based protein phylogeny. Large terminase nodes and groups are colored based on phage DNA packaging mechanisms (Casjens *et al*., 2005). Phages in the VpV262-like group cluster.

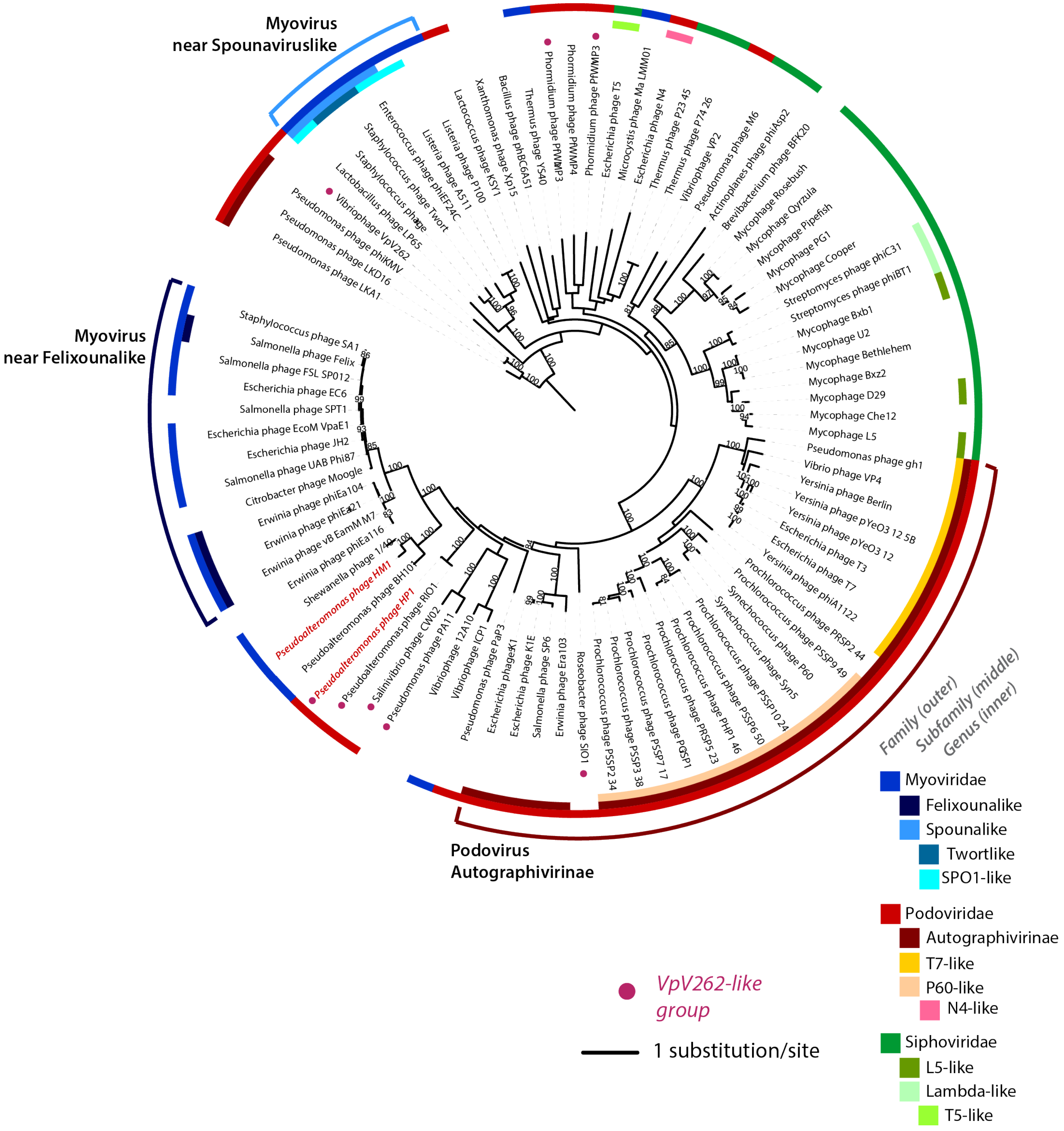

Supplementary Figure 5. DNA polymerase A (DNApol) maximum likelihood-based protein phylogeny. Colorblocks indicate phage taxonomy and groups are colored based on phage DNA packaging mechanisms (Casjens *et al.,* 2005). Phages in the VpV262-like group are spread across the tree.

Supplementary Material

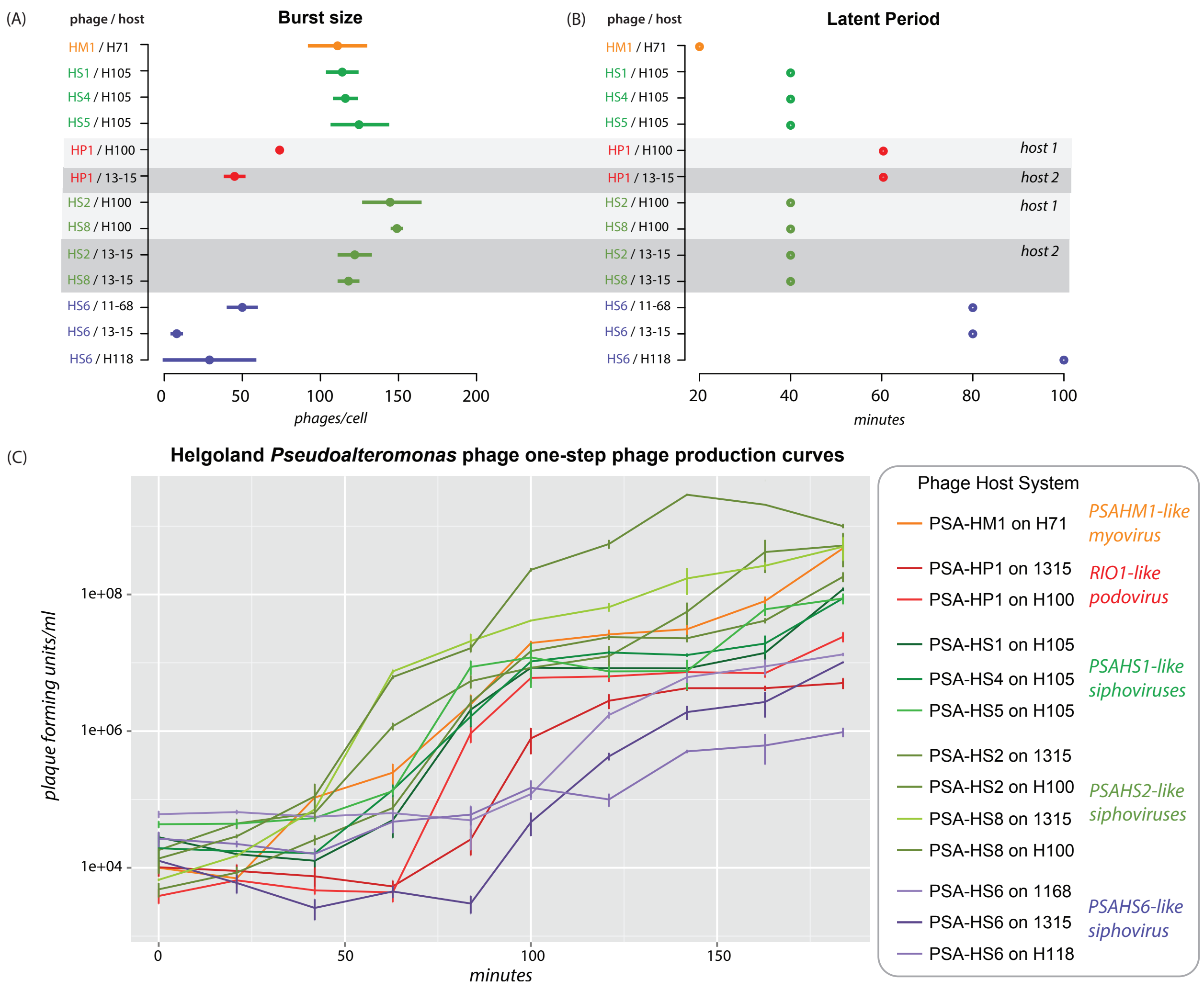

Supplementary Figure 6. Infection phenotype data for Helgoland *Pseudoalteromonas* phages and hosts. **(A)** Burst sizes, **(B)** latent periods, **(C)** one-step virus production curves.

Supplementary Table 1 (data sheet 2.csv). VirSorter output describing prophages predicted to be in *Pseudoalteromonas* phage genomes and WGS projects in GenBank. These identified prophages were used in the reciprocal best blast used in Figure 1 and Supplementary Figure 1.

Supplementary Table 2 (data sheet 3.xlsx). Annotation tables for all newly sequenced Helgoland phage isolates.

Supplementary Table 3 (data sheet 4.xlsx). Summary of mass spectroscopy-derived proteomic data for each protein (CDS) of each Helgoland phage isolate analyzed, including data for input to R script to recreate model SDS-PAGE gel in Figure 3.

Supplementary Material

Methods for Supplementary Information:

Gene phylogenies

Full-length 16S rRNA gene sequences were recovered from the sequenced host genome data using EMIRGE (Miller *et al*., Banfield, 2011). Alignment for the 16S rRNA gene phylogeny was generated using the SINA aligner (Pruesse *et al.*, 2012). Alignments for terL and DNApol protein phylogenies were generated with T-Coffee (Notredame *et al.*, 2000) with default parameters. Maximum likelihood trees were generated with RaXML v 7.3.0 based on 100 bootraps and using the Generalized Time Reversible gamma model (Stamatakis 2006). Trees visualizations were generated in iTOL v2 (Letunic and Bork, 2016).

References for Supplementary Information:

1. Casjens, S. R., Gilcrease, E. B., Winn-Stapley, D. A., Schicklmaier, P., Schmieger, H., Pedulla, M. L., Ford, M.E., Houtz, J.M., Hatfull, G.F., Hendrix, R. W. (2005). The generalized transducing salmonella bacteriophage ES18: Complete genome sequence and DNA packaging strategy. *J. Bacteriol.* **187**, 1091-104.
2. Letunic, I., Bork, P. (2016). Interactive tree of life (iTol) v3: An online tool for the display and annotation of phylogenetic and other trees. *Nucleic Acids Res.* **44**, W242-5.
3. Miller, C. S., Baker, B. J., Thomas, B. C., Singer, S. W., Banfield, J. F. (2011). EMIRGE: Reconstruction of full-length ribosomal genes from microbial community short read sequencing data. *Genome Biol*. **12**, R44.
4. Notredame, C., Higgins, D. G., Heringa, J. (2000). T-Coffee: A novel method for fast and accurate multiple sequence alignment. *J. Mol. Biol.* **302**, 205-217.
5. Pruesse, E., Peplies, J., Glöckner, F. O. (2012). SINA: Accurate high-throughput multiple sequence alignment of ribosomal RNA genes. *Bioinformatics*, **28**, 1823-1829.
6. Stamatakis, A. (2006). RAxML-VI-HPC: Maximum likelihood-based phylogenetic analyses with thousands of taxa and mixed models. *Bioinformatics*, **22**, 2688-2690.
7. Wiederstein, M., Sippl, M. J. (2007). ProSA-web: Interactive web service for the recognition of errors in three-dimensional structures of proteins. *Nucleic Acids Res.* **35**, W407-10.
